# A New Type of Air Conditioning System Based on Finned Ceiling Radiant Coupled with Independent Fresh Air and Its Thermal Comfort Experimental Study

**DOI:** 10.1155/2022/4144569

**Published:** 2022-09-17

**Authors:** Wenqi Qin, Yingning Hu, Jinwen Su, Yubang Hu

**Affiliations:** ^1^College of Civil Engineering and Architecture, Guangxi University, Nanning 530004, China; ^2^School of Mechanical Engineering, Guangxi University, Nanning 530004, China

## Abstract

The traditional radiation air conditioning system has some problems, such as easy condensation, insufficient refrigeration capacity, complex structure, and control system. Therefore, this study proposes a new type of finned metal radiant plate with large heat flow per unit area, sufficient cooling capacity, and simplified heat exchange system, in order to realize large temperature difference between cooling and heating. The temperature field uniformity and thermal comfort test of a novel type of finned ceiling radiant panel and independent fresh air linked air conditioning system under summer cooling and winter heating circumstances are accomplished through artificially generated climate environments. The study's findings demonstrate that in the radiation and fresh air modes, the maximum interior temperature differential under cooling conditions does not rise over 2.1°C. The maximum temperature differential in the space at any one moment in the radiation and fresh air modes cannot be greater than 3°C when heating conditions are present. The fresh air's cooling and dehumidifying effects are clear. The dehumidification efficiency may reach 50%, and the moisture content ranges from 5.48 to 9.63 g/kg. With PMV ranging from −0.34 to 0.54, the enhanced air conditioning system in this research provides exceptionally good thermal comfort. Additionally, the finned radiant panel's installation area occupies just 14% of the ceiling, which is sufficient to fulfill the room's cooling and heating load needs as well as provide high thermal comfort and consistent indoor temperature. The theoretical investigation and practical implementation of the direct expansion radiant air conditioning system are both strongly supported by this research.

## 1. Introduction

Radiant air conditioners have attracted more and more attention because of their good thermal comfort. In recent years, many experts and scholars have conducted a lot of research work on the performance of radiant air conditioners. In terms of the theoretical model and simulation research of radiant air conditioning, Wu et al. [1] proposed and established a new simplified model to calculate the surface temperature and heat flux of the floor radiant heating and cooling system and verified the model with the measured data. Using a radiative heat transfer model, Bahadori et al. [2] developed a prediction tool to estimate the characteristic temperature ratio of a plate radiator and a system of heat exchange plates with a high temperature. The computation approach is demonstrated to be in good agreement with the observed data by comparison, and the average absolute variation is just approximately 0.2%. Tang et al. [3] derived an analytical expression for the dynamic thermal response of a radiant floor slab under variable solar radiation using a new approximate harmonic method. The results show that the dynamic heat transfer process calculated by the new approximate harmonic method is in good agreement with the 3D model. Based on three radiation models, Lv et al. [4] established a synchronous thermal model to calculate the indoor air temperature and the indoor wall temperature. Merabtine et al. [5] proposed a new analytical correlation-based simplified model to evaluate the surface temperature of a heat exchange plate and study its thermal performance under dynamic conditions. Finally, numerical models based on the finite difference method and finite volume method are established. Yu et al. [6] established a simplified model of top-insulated metal ceiling radiant cooling panels (CRCP-s) using a serpentine arrangement to predict the average temperature of the panels, outlet water temperature, and cooling capacity. Experiments were carried out on two CRCP-s, and the results showed that the outlet water temperature and cooling capacity predicted by the model were in good agreement with the experimental measurements. By introducing an inner air layer into the improved ceiling radiant cooling panels (CRCP) system, Su et al. [7] established a computational model for the heat transfer analysis of the improved CRCP system and verified it experimentally. The results show that thicker aluminum plates, thicker heat exchange plates, and thinner air layers increase cooling capacity. Zhao et al. [8] used the CFD model of the exposed capillary ceiling radiant panel (E-CCRP) system office to analyze the heat transfer characteristics and time-delay characteristics of the E-CCRP winter start-up process under unsteady conditions, and experiments are used to confirm that the simulation findings are accurate. Khatri et al. [9] performed computational fluid dynamics (CFD) simulations using ANSYS to determine the thermal performance of the radiative cooling system. Moreover, the air space distribution of the radiation system and the conventional system is simulated by the computational fluid dynamics (CFD) method.

In addition, many researchers have carried out a lot of research work on the performance of radiant air conditioners. Based on the indoor comfort and energy consumption of a demonstration office building, Zhang et al. [10] analyzed the operating conditions and key energy-saving technologies of the compound air conditioning system and conducted a large number of experimental tests and accurate simulations. Research has confirmed that the ground source heat pump combined with the floor radiant air conditioning system is more energy efficient. Du et al. [11] conducted a numerical study on the influence of the changes in the design and operating parameters of the heat exchange plate on indoor thermal comfort and energy-saving performance in the human sleep environment. The results show that the four operating parameters have significant effects on bedroom thermal comfort and energy-saving performance when the R-TAC system is used. Ding et al. [12] made a simplified calculation of the heating capacity, heat loss, and surface temperature of a radiant heating floor with a high-performance thermal insulation layer and verified it with measured data and numerical simulation data. Sun et al. [13] proposed a new type of flat heat pipe radiant heating and cooling integrated terminal and studied the thermal performance (including thermal response speed, heating and cooling capacity, heating and cooling temperature, and heat transfer coefficient) of the flat heat pipe terminal through experiments. Lv et al. [14] developed a new type of heat sink, which consists of an upper flat plate, a lower groove plate, and a heat transfer liquid filling the gap between the two plates. Tang et al. [15] carried out an experimental study on the droplet size from superhydrophobic surfaces and conventional aluminum alloy surfaces. Jin et al. [16] studied the condensation characteristics of the heat exchange plate through experiments and simulations. The temperature of the heat exchange plate and the relative humidity of the adhesion layer during the start-up phase were tested, and their effects on the setting time were analyzed. Tang et al. [15] performed a theoretical study of condensation rates using a simplified N–S approximation equation and derived a heat and mass transfer analogy. In the experiments, the condensation rates of different lengths of radiant cooling panels were measured at different locations, including floors, walls, and ceilings.

In recent years, some scholars have carried out some researches on the indoor environment of radiant air conditioning systems. Su et al. [17] conducted experiments on radiator heating and floor heating in a controlled climate room. The experiment recruited 66 college students as volunteers to investigate their physical and psychological responses. Wang et al. [18] recruited 20 volunteers to participate in thermal comfort experiments in 2 nonuniform environments and used questionnaires for subjective evaluations. Chao et al. [19] recruited 30 experiencers to participate in a questionnaire survey under 8 different experimental conditions under the fan coil and floor radiant cooling system and collected the indoor thermal environment parameters of the 2 systems at different heights through instrument monitoring. Tian et al. [20] conducted a field study of occupant thermal comfort and the thermal environment with a heat exchange slab cooling system, collecting 116 sets of data from 82 participants during summer and winter. For the segmental thermal interaction between the human body and the surrounding environment, Atmaca et al. [21] modified the Gagge-2 node model appropriately to simulate the multisegment situation and verified the local differences.

With the increasing maturity of artificial intelligence technology, artificial intelligence has been widely used in various fields, and many scholars have put forward many models and control algorithms in the field of artificial intelligence. Zhou et al. [22] proposed a fuzzy adaptive synchronization control algorithm with a composite learning technique for a class of incommensurate fractional-order chaotic systems with mismatched parametric uncertainties based on T-S fuzzy models presented. Zhou et al. [23] studied a generalized function projective synchronization of incommensurate fractional-order chaotic systems with inputs saturation. In the study of Zhou et al. [24], passive, active, and combined passive active solutions in PCMs systems have been comprehensively reviewed when being applied in heating, cooling, and electrical systems, together with a dialectical analysis of advantages and disadvantages. In another study by Zhou et al. [24], machine learning methods are effective to assist the energy-efficient renewable systems during multicriteria design and multilevel uncertainty-based operation periods. Zhou et al. [24] pointed out in the study that machine learning methods are promising for thermal and energy performance improvement, through surrogate model development, model predictive control, and optimisation function. Using appropriate intelligent control methods in the HVAC system not only can improve the efficiency of the system and reduce the energy consumption of the system but also can create a good thermal comfort environment for people. In the heating system, model predictive control (MPC) is an advanced control technology. Siroky et al. [25] found that its energy-saving potential in the weather forecast of building heating systems is between 15% and 28%. Shafiei et al. [26] proposed a predictive control model to directly control the power consumption of large refrigeration systems. Beghi et al. [27] designed a reinforcement-learning algorithm that learns to operate a cooling system based on its interaction with the environment. The above methods improve the efficiency of the refrigeration system by 20%∼30% and reduce the wear of various components.

Aiming at the problems existing in the application of traditional radiant air conditioners, this study proposes and manufactures a new type of direct-expansion ceiling radiant and independent fresh air coupled air conditioning system. The system uses R410A as the refrigerant and uses the self-designed finned metal radiant plate structure, which can effectively prevent condensation and solve the problem of insufficient cooling capacity. In the artificial simulated climate environment, the system operating characteristics of the radiant fresh air mode under the conditions of low-temperature heating in winter, normal heating in winter, normal cooling in summer, and high-temperature cooling in summer are studied. Finally, the indoor temperature distribution uniformity, cooling effect, and dehumidification effect of the system under various working conditions are studied through experiments, and then the indoor thermal comfort index (predicted mean vote-predicted percentage dissatisfied (PMV-PPD)) value is calculated. In conclusion, the work and significance of this study are as follows.

Aiming at the shortcomings of the traditional radiant air conditioning system with water as the medium, this paper designs a new radiant air conditioning system based on variable air volume (VAV), which is called a frequency conversion control air conditioning system with a roof radiation module and independent fresh air function. In the system developed in this study, the roof radiation module and the independent fresh air system directly use the refrigerant R410 as the cold medium and are driven by the frequency conversion operation of the compressor, so as to simplify the radiation air conditioning system and make the radiation air conditioning system obtain accurate control. The radiant cooling and radiant heating share a radiant roof end system and add an independent fresh air system to improve indoor comfort proposed in this study, which provides some design theory support and practical value for the application of direct expansion radiation air conditioning system.

In this paper, aiming at the problems of small cooling capacity and easy condensation at the radiation end of the traditional radiation air conditioning, we designed a finned metal radiation plate. The finned metal radiation plate has inlined fins. The aluminum fins increase the contact area with the air, strengthen the heat transfer ability of the copper tube, and increase the convective heat transfer ability of the radiation plate. In addition, the working medium in the copper tube is R410 A, and the groove-type baffle plate discharges the condensed water generated under the refrigeration condition. Our design of finned metal radiant plate provides a reference for the promotion, development, and application of inlined finned structure radiant plate.

This paper takes the direct-expansion roof radiation and independent fresh air coupling air conditioning system as the research objective. Moreover, we analyze the dehumidification effect of the system under refrigeration and dehumidification conditions by testing the distribution uniformity of the indoor temperature field and calculate the indoor thermal comfort index (predicted mean vote-predicted percentage dissatisfied (PMV-PPD)) value, which provides theoretical and application reference for the research and development of this kind of air conditioning system.

## 2. Design of Air Conditioning System Coupled with Direct Expansion Ceiling Radiation and Independent Fresh Air

### 2.1. Finned Metal Radiant Panel

**Figure 1:**
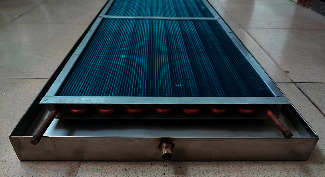
Finned metal radiant panel.

**Figure 2:**
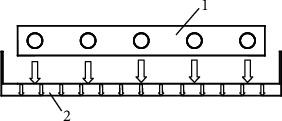
Schematic diagram of heat transfer of finned metal radiant plate. 1 – the finned radiant plate and 2 – grooved shielding plate.

Figure 1 depicts the genuine finned metal radiant panel. As can be seen from the figure, it is different from the traditional suspended radiant panel. The finned metal radiant panel has fins arranged inline, which can increase the contact area with the air, strengthen the heat transfer capacity of the copper tube, and increase the convective heat transfer capacity of the radiant panels. The medium in the copper tube is R410A, and the groove-type shielding plate drains the condensed water generated under refrigeration conditions. The groove-type shielding plate is made of stainless steel and has a built-in thermal insulation material to reduce the thermal conductivity of the shielding plate, which can reduce the risk of condensation on the lower surface. The schematic diagram of heat exchange of finned metal radiant plate is shown in Figure 2. It can be seen from Figure 2 that the lower surface of the entire finned radiant plate transfers the cooling down. Due to the temperature difference between the upper and lower surfaces of the air interlayer, natural convection and radiation heat transfer will occur in the air interlayer. The cooling energy is transferred to the upper surface of the groove-type shielding plate; then the upper surface of the rear groove-type shielding plate is transferred to the lower surface through heat conduction; and finally, the lower surface of the groove-type shielding plate participates in the heat exchange of the indoor environment.  Table 1 lists the structural parameters of the finned metal radiant panels.

### 2.2. Air Conditioning System Coupled with Direct Expansion Ceiling Radiation and Independent Fresh Air

**Figure 3:**
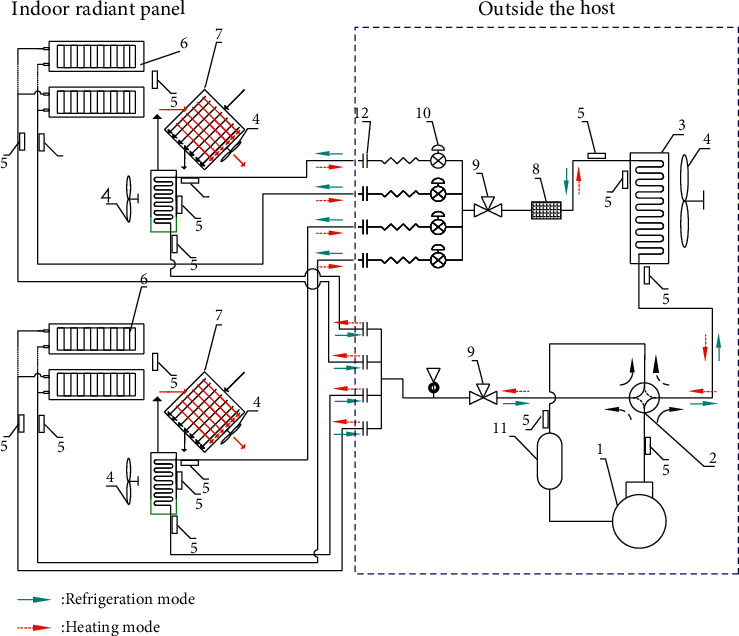
Schematic diagram of the direct-expansion ceiling radiation and independent fresh air coupled air conditioning system. 1 – compressor, 2 – four-way reversing valves, 3 – heat exchanger, 4 – ventilation fan, 5 – temperature sensor, 6 – radiation terminal, 7 – heat exchange core, 8 – filter, 9 – main service valve, 10 – electronic expansion valve, and 11 – liquid storage tank.

In this study, the self-designed direct-expansion ceiling radiation and independent fresh air coupling air-conditioning system adopts the form of one machine and multiple connections. It is made up of a radiant terminal, a fresh air ventilator, and a heat pump air conditioner host, as can be seen in Figure 3. The radiation terminal is composed of four finned heat exchange plates connected in series, which have the characteristics of a compact structure and a large heat exchange area. The compound air conditioning system directly uses refrigerant R410 A as a heat transfer medium to heat or cool the air. In addition, the fresh air ventilator adopts the direct expansion freezing and dehumidification method, which can obtain a better dehumidification effect. Compared with the traditional radiant air-conditioning system using water as the medium, the air-conditioning system proposed in this study no longer needs a complex water mixing center and a dew point monitoring system because it does not require a special waterway system, so it has some advantages, including equipment small footprint, simple structure, easy control, convenient inspection, and maintenance. In addition, it has the advantages of energy saving and environmental protection, shortened response time, and avoidance of condensation.

The coupled air conditioning system designed in this study can avoid cold condensation dew, mainly for two reasons. First, the fresh air system adopts direct expansion and freezing dehumidification, and the fresh air is sent indoors after dehumidification so that the fresh air can be treated to avoid condensation. Second, each finned metal radiation plate is equipped with a groove-type shielding plate below, which is to prevent condensation and discharge condensate when refrigeration; even if the radiation plate condensation, the groove-type shielding plate can receive the drops of water falling on the surface of the finned heat exchange plate and discharge through the drain pipe.

## 3. Experimental Platform

### 3.1. Introduction of the Experimental Platform

**Figure 4:**
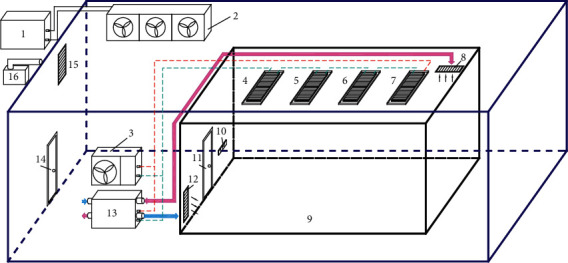
Schematic diagram of the experimental platform of this study. 1 – outdoor environment host, 2 – indoor machine for the outdoor environment, 3 – heat pump air conditioning unit, 4–7 – finned metal radiant panels, 8 – return air outlet, 9 – indoor environment test room, 10 – control panel, 11 – inner door, 12 – fresh air outlet, 13 – fresh air ventilator, 14 – outer door, and 15 – humidifier outlet.

Experimental platform structure diagram is shown in Figure 4. The experimental platform is mainly composed of two parts, one is outdoor, which simulates climate change using an artificial climate chamber. Its spatial structure size (length, width, and height) is 7.7 m × 5.7 m × 3.5 m. The other part is the room, which is used to test the effect of indoor heating, refrigeration, and fresh air exchange. Its spatial structure size (length, width, and height) is 4.16 m × 4.06 m × 3 m. The outdoor environment can be controlled by temperature and humidity (the temperature adjustment range is −20°C to 55°C, and the relative humidity of the outer room can be adjusted to more than 90%). In order to ensure that the experimental room in this study has good thermal insulation, the envelope structure of the inner room adopts polyurethane thermal insulation material.

### 3.2. Arrangement of Finned Metal Radiant Panels

**Figure 5:**
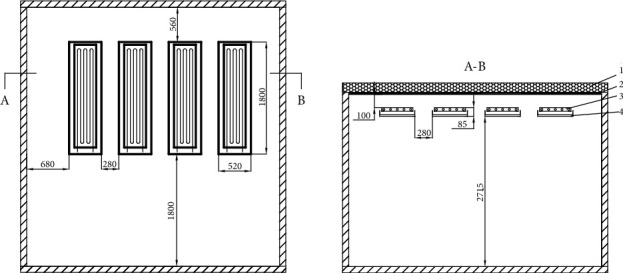
Plan and sectional view of radiant panel arrangement. 1 – thermal insulation layer, 2 – reflective layer, 3 – finned radiant plate, and 4 – groove-type shielding plate.

The distribution of the four finned radiant panels on the ceiling of the room in the experiment is shown in Figure 5. The radiant panel is 100 mm away from the top of the ceiling. It can be seen from its sectional view that 1 is the thermal insulation layer, which can prevent the loss of cooling capacity. A layer of aluminum foil is attached under the heat insulation layer as a reflective layer (marked as 2 in Figure 5) to reflect the radiant cooling from the finned radiant plate. Number 3 is a finned metal radiant plate, which exchanges heat to the indoor environment in the form of natural convection and radiation heat transfer. Number 4 in Figure 5 is a groove-type shielding plate, which can collect the dew condensation water of the radiant panel. There is a heat insulation layer inside the groove-type shielding plate, which plays a buffering role in energy conduction, so that the energy conduction in the room is uniformly distributed. In the artificial simulated climate chamber designed in this study, the total area of the four finned metal radiant panels is 2.38 m^2^; the total area of the inner room ceiling is 16.89 m^2^; and the installation area of the finned metal radiant panels only accounts for 14% of the ceiling.

### 3.3. Experimental Method

In the experiment of this study, by changing the outdoor ambient temperature, the system is set to four operating conditions, including low-temperature heating, normal heating, normal cooling, and high-temperature cooling, each of which have different temperature ranges. The indoor space test point of this experiment is to measure the temperature, humidity, and wind speed of three planes, which are *Z* = 0.1 m (the position of the human ankle), *Z* = 1.1 m (head position when sitting), and *Z* = 1.7 m (head position when standing). Finally, according to the measurement data of temperature, humidity, and wind speed, it is quantitatively determined what the PMV-PPD value is for interior cooling and heating situations. The time interval for collecting experimental data in this study was 1 min. In order to ensure the accuracy of the experimental data, each plane adopts the method of multipoint measurement and averaging, thereby minimizing the error.

### 3.4. Test Parameters and Experimental Apparatus

The test instruments used in this study include a multichannel paperless recorder, a temperature, and humidity transmitter, a PT100 temperature sensor, an automatic temperature and humidity recorder, and an anemograph. The parameters of each instrument are shown in Table 2.

### 3.5. Location Distribution of Experimental Test Points

**Figure 6:**
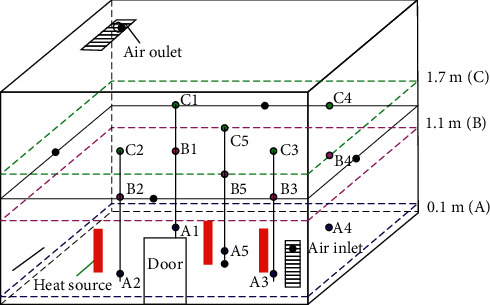
Sensor layout diagram in the room.

In this paper, the indoor thermal comfort of the composite air conditioning system under artificial simulated climate conditions is studied. It mainly analyzes the indoor temperature field distribution, so it is necessary to arrange temperature and humidity sensors in different parts of the room. In the experimental design, the sensors are arranged in different places, including the surrounding walls of the room, the center of the ceiling and the floor, as well as the air inlet and outlet. In addition to the enclosure structure, it is also necessary to install some sensors for the three planes of *z* = 0.1 m, *z* = 1.1 m, and *z* = 1.7 m in the indoor space and ensure that five sensors are installed on each surface. A schematic diagram of the arrangement of the temperature and humidity sensors in three planes is shown in Figure 6.

The specific position coordinates of the test points in the indoor space are shown in Table 3.

### 3.6. Experimental Working Condition

All the experimental instruments must be calibrated before the experiment begins. At the beginning of each experiment, firstly turn on the air conditioner of the outer room to make the temperature of the outer room reach the setting range of the corresponding working condition and then open the door of the inner room to keep the temperature of the inner room and the outer room as the same as possible. Finally, the door to the inner room needs to be closed.  Table 4 is the specific experimental scheme of this study.

## 4. Experimental Results and Analysis

Each working condition runs for 24 hours, and each test point's temperature and humidity are automatically recorded every minute. Finally, an experimental curve is drawn based on data with a sampling interval of half an hour for analysis. Before each working condition test, turn on the air-conditioning equipment in the artificial climate environment laboratory to make the outdoor simulated temperature reach the set temperature range. Open the door of the inner room before the test so that the initial indoor temperature is basically the same as the temperature of the artificial simulated outdoor environment. After closing the door of the inner room, the system operation mode is turned on, and the temperature and humidity changes in the room are recorded. This paper mainly studies the temperature distribution of five test points on the *z* = 0.1 m, *z* = 1.1 m, and *z* = 1.7 m horizontal planes.

### 4.1. Variation of Indoor Temperature Field under Cooling Conditions

#### 4.1.1. Common Refrigeration Conditions

The experimental conditions of ordinary refrigeration conditions are set as the outdoor environment simulation temperature is 28°C∼30°C, the relative humidity is 75%, the indoor heat source is set at 300 W, and the indoor air conditioning temperature is set at 25°C. The heat source arrangement is shown in Figure 6.

**Figure 7:**
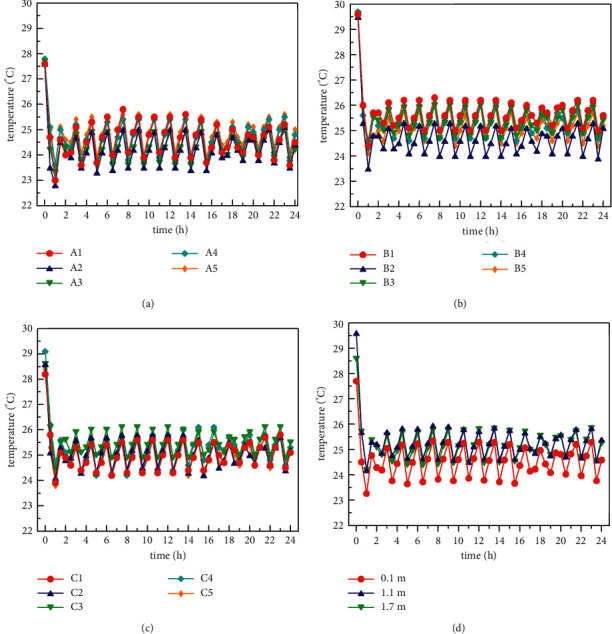
Indoor temperature distribution under normal refrigerating condition (28°C∼30°C): (a) *Z* = 0.1 m horizontal temperature distribution, (b) *Z* = 1.1 m horizontal temperature distribution, (c) *Z* = 1.7 m horizontal temperature distribution, and (d) average temperature distribution of each horizontal plane.

When the radiation and fresh air modes are turned on at the same time in the normal cooling mode in summer (the outdoor simulated temperature is 28°C∼30°C), the temperature of the three indoor horizontal surfaces and the average temperature distribution of each horizontal surface are shown in Figure 7. Figures 7(a)–7(c) show that the temperature changes in the three horizontal planes follow the same pattern. This pattern is demonstrated by the fact that the indoor temperature drops to the set temperature (25°C), the system is turned off for an hour, and then the indoor temperature rises. When the indoor temperature exceeds 25°C, the air conditioning unit will start again. After about half an hour of operation, the indoor temperature will drop below 25°C. Then the air conditioning unit was shut down again for 1 hour. The air conditioning units of the air conditioning system designed in this study basically start and stop according to this rule. After the system is turned on for about 2 hours, it reaches a steady state. During the test time after the system is stabilized, the temperature in the room is kept between 23.4°C and 26.3°C. When the whole system is in a steady state, the temperature difference of the horizontal surface of *z* = 0.1 m is within 0.9°C; the temperature difference of the horizontal surface of *z* = 1.1 m is within 1.1°C; and the temperature difference of the horizontal surface of *z* = 1.7 m is within 0.9°C at the same time. In addition, the maximum difference in temperature of the vertical plane is within 1.8°C. The above temperature distribution characteristics indicate that in the human activity area, the temperature distribution of the indoor space is relatively uniform.

#### 4.1.2. High-Temperature Refrigeration Conditions

In this study, under the condition of high-temperature refrigeration, the experimental settings are that the simulated outdoor temperature is 37°C∼39°C, the relative humidity is 80%, the indoor heat source is set at 300 W, and the indoor air conditioning temperature is 25°C.

**Figure 8:**
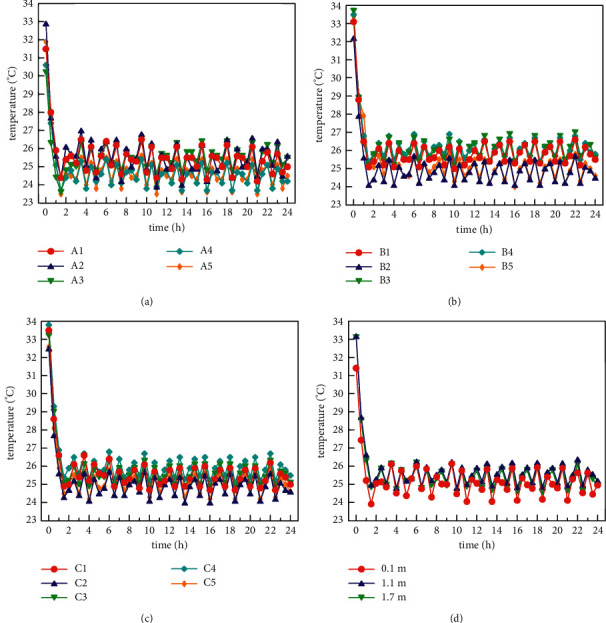
Indoor temperature distribution under high-temperature refrigeration condition (37°C∼39°C): (a) *Z* = 0.1 m horizontal temperature distribution, (b) *Z* = 1.1 m horizontal temperature distribution, (c) *Z* = 1.7 m horizontal temperature distribution, and (d) average temperature distribution of each horizontal plane.

When the radiation and fresh air systems are opened at the same time, the distribution of the temperature in the three indoor horizontal planes and the average temperature in each horizontal plane under the condition of high-temperature refrigeration in summer (outdoor simulated temperature ranges from 37°C to 39°C) is shown in Figure 8. It can be seen from Figures 8(a)–8(c) that the temperature variation trend of the three horizontal planes is consistent. It is shown that the indoor temperature drops below the preset temperature (25°C) about 1 hour after the system is turned on, and then the system is shut down for 1 hour, followed by the indoor temperature rise. When the indoor temperature exceeds 26°C, the air conditioning unit will start again. After the equipment works for about half an hour, the indoor temperature drops to below 24.5°C again, and then the air conditioning unit shuts down again for 1 hour. The air conditioning unit of the whole air conditioning system basically starts and stops in accordance with this rule. After about 3 hours, the whole system reaches a steady state. After the system is stable, the indoor temperature is kept between 23.6°C and 26.6°C. According to the test results, at the same time, the horizontal plane temperature difference of *Z* = 0.1 m is kept within 1.5°C; the horizontal plane temperature difference of *Z* = 1.1 m is kept within 1.5°C; and the horizontal plane temperature difference of *Z* = 1.7 m is kept within 1.3°C. It can be seen from Figure 8(d) that the maximum temperature difference in the vertical plane is within 2.1°C. The above experimental analysis results show that the indoor temperature distribution of the system is relatively uniform under the high-temperature refrigeration condition.

### 4.2. Variation of Indoor Temperature Field under Heating Conditions

#### 4.2.1. Normal Heating Condition

In the test experiment of this study, the experimental settings under the normal heating condition are that the outdoor environment simulation temperature is 1°C∼5°C, the relative humidity is 30%, and the indoor air conditioner setting temperature is 26°C.

**Figure 9:**
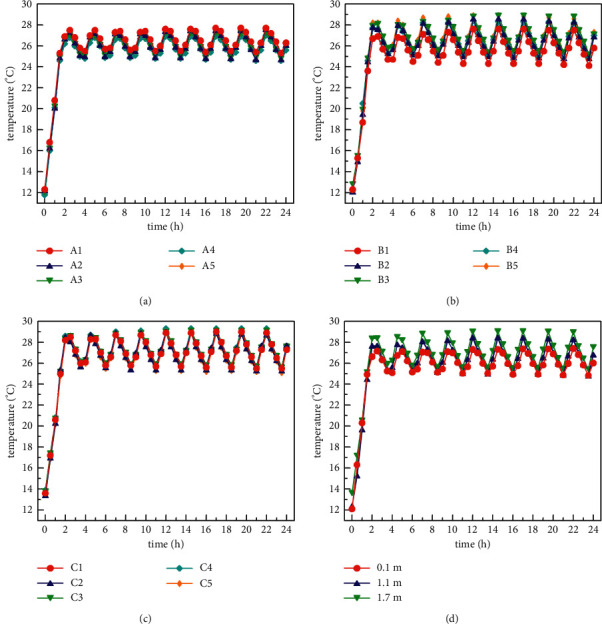
Indoor temperature distribution under normal heating conditions (1°C∼5°C): (a) *Z* = 0.1 m horizontal temperature distribution, (b) *Z* = 1.1 m horizontal temperature distribution, (c) *Z* = 1.7 m horizontal temperature distribution, and (d) average temperature distribution of each horizontal plane.

In this experiment, when the radiation and fresh air modes are turned on at the same time, the distribution of indoor temperature in three horizontal planes and the average temperature in each horizontal plane under the conditions of ordinary heating in winter (outdoor simulated temperature ranges from 1°C to 5°C) is shown in Figure 9. From Figures 9(a)–9(c), it can be seen that the experimental system of three horizontal surface temperature changing trends is consistent, if the indoor temperature exceeds the preset temperature (26°C) after two hours of continuous operation, the system automatically stops working for two hours, then the system automatically stop 2 hours, then the indoor temperature drops. When the indoor temperature is lower than the preset temperature, the air conditioning unit will start the heating work again. After about 1 hour of work, the indoor temperature exceeds the preset temperature again, and then the air conditioning unit shuts down again for 1.5 hours. In the subsequent experimental test, the air conditioning unit basically starts and stops working in accordance with this rule. In the above working cycle, the air conditioning unit is turned on for 1 hour and shut down for 1.5 hours. It takes two hours for the system to reach a steady state and start recording data. Recorded experimental data show that the temperature in most areas of the room is always maintained between 25°C and 28°C. In the steady state of the system, the horizontal plane temperature difference of *Z* = 0.1 m at the same time is within 0.9°C; the horizontal plane temperature difference of *Z* = 1.1 m is within 1.5°C; and the horizontal plane temperature difference of *Z* = 1.7 m is within 1°C. In addition, the maximum temperature difference in the vertical plane of the interior space is within 2.4°C. The above experimental analysis results show that the indoor temperature distribution of the system is relatively uniform under the normal heating condition.

#### 4.2.2. Low-Temperature Heating Condition

In the low-temperature heating condition of this study, the experiment is set as the simulated outdoor environment temperature of [−10°C, −6°C], relative humidity of 30%, and indoor air conditioning temperature of 26°C.

**Figure 10:**
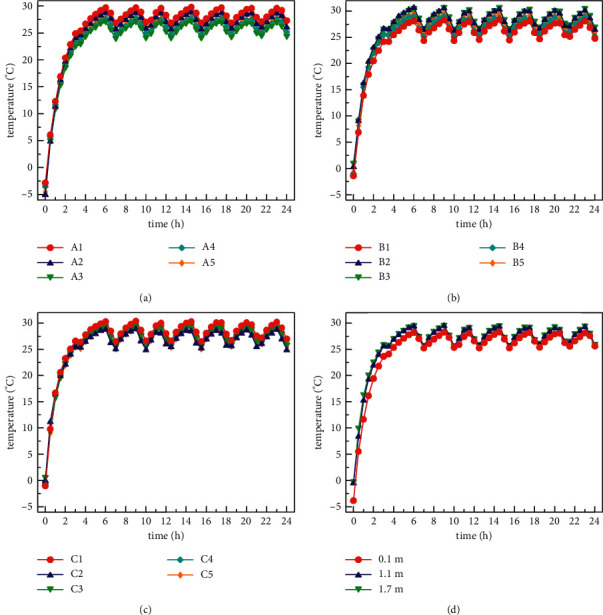
Indoor temperature distribution under low-temperature heating condition (–10°C∼–6°C): (a) *Z* = 0.1 m horizontal temperature distribution, (b) *Z* = 1.1 m horizontal temperature distribution, (c) *Z* = 1.7 m horizontal temperature distribution, and (d) average temperature distribution of each horizontal plane.

When the radiation and fresh air modes are opened at the same time, the distribution of indoor temperature in three horizontal planes and the average temperature in each horizontal plane under the conditions of low-temperature heating in winter (outdoor simulated temperature ranges −10°C to −6°C) is shown in Figure 10. It can be seen from Figures 10(a)–10(c) that the temperature variation trend of the three horizontal planes is consistent. The working process of the air conditioning system is that the indoor temperature rises above the preset temperature (26°C) about three hours after the system is turned on. Then the system shuts down for an hour, and the temperature drops. When the indoor temperature is lower than the preset temperature, the air conditioning unit will start again. After the system equipment works for about 1 hour, the indoor temperature rises to above 26°C again, and then the air conditioning unit shuts down again for 1 hour. In the subsequent experimental test, the air conditioning unit basically starts and stops working in accordance with this rule. The air conditioning system works for 1 hour and stops for 1 hour in a cycle. The air conditioning system proposed in this study reaches a steady state after about 5 hours, and the temperature in most areas of the room always stays between 27°C and 30°C after the system is stabilized. In the laboratory, the temperature difference of the inner room in the horizontal plane *z* = 0.1 m is within 2.9°C, in the horizontal plane *z* = 1.1 m is within 2.4°C, and in the horizontal plane *z* = 1.7 m is within 1.9°C. In addition, the maximum temperature difference in the vertical plane of the inner room is within 3°C. In conclusion, the experimental test results of the air conditioning system designed in this study show that the indoor temperature distribution is relatively uniform under working conditions of low-temperature heating.

## 5. Dehumidification Efficiency Analysis of Fresh Air System

Since dehumidification process exists in both the refrigeration condition and the fresh air system, this study analyzed the dehumidification efficiency of the fresh air system in three working conditions: ordinary fresh air refrigeration (the temperature range is 31°C to 33°C), high-temperature refrigeration (the temperature range is 37°C to 39°C), and dehumidification in a transition season (the temperature range is 20°C to 22°C). The moisture content of outdoor air and indoor air under the above three working conditions is shown in Table 5.

It can be seen from Table 5 that the average moisture content of outdoor air and indoor air is 11.33 g/kg and 5.48 g/kg under dehumidification conditions in the transition season, indicating that the average dehumidification efficiency of the fresh air system is 51.6% under such conditions. The average moisture content of outdoor air and indoor air is 22.91 g/kg and 8.9 g/kg under ordinary fresh air refrigeration, indicating that the average dehumidification efficiency of the fresh air system is 61.2% under such conditions. The average moisture content of outdoor air and indoor air is 32.6 g/kg and 9.63 g/kg under high-temperature refrigeration, indicating that the average dehumidification efficiency of the fresh air system is 70.4% under such conditions. The experimental results show that the average dehumidification efficiency is more than 50% under the above three working conditions, which is sufficient to prove that the fresh air system designed in this study has a good dehumidification effect.

## 6. Thermal Comfort Analysis

### 6.1. PMV-PPD

The factors affecting thermal comfort can be divided into two categories: one is environmental factors, including air temperature, airflow velocity, partial pressure of water vapor in the air, and average radiant temperature. Another category is human factors, including human metabolic rate and thermal resistance of clothing. Professor Fanger expanded the thermal comfort equation and the calculation formula of PMV through the data of many subjects. The equation for calculating PMV is

(1) $$\begin{array}{c} \text{PMV}=\left(0.303e^{-0.036M}+0.028\right)\\ \times \left\{\left(M-W\right)-3.05\times 10^{-3}\times \left[5733-6.99\left(M-W\right)-P_{a}\right]-0.42\times \left[\left(M-W\right)-58.15\right]-1.7\times 10^{-5}M\left(5867-P_{a}\right)-1.4\times 10^{-3}M\left(34-t_{a}\right)-3.96\times 10^{-8}f_{cl}\times \left[{\left(t_{cl}+273\right)}^{4}-{\left(t_{r}+273\right)}^{4}\right]-f_{cl}\cdot h_{c}\cdot \left(t_{cl}-t_{a}\right)\right\}, \end{array}$$

where *M* is metabolic rate, Met; *W* stands for human body power, W/s; *P*_*a*_ is the partial pressure of water vapor in the air, Pa; *t* is the air temperature, °C; *f*_*cl*_ is the ratio of the surface area of the clothed body to the naked body; *t* is the mean radiation temperature, °C; *h*_*c*_ is the convective heat exchange coefficient, W/(s·m2°C); and *t*_*cl*_ is the average temperature of the outer surface of the wearer's body, °C.

When the PMV value is determined, the PPD value can be calculated by the following formula:

(2) $$\begin{array}{c} \text{PPD}=100-95\;\exp\left[-\left(0.03353\times PMV^{4}+0.2179PMV^{2}\right)\right]. \end{array}$$

### 6.2. Indoor Thermal Comfort Analysis

When the indoor temperature and humidity change is basically stable after the air conditioning unit runs stably, the thermal comfort index PMV-PPD value of the indoor *z* = 1.1 m plane is calculated. The calculation results of thermal comfort are shown in Table 6.

It can be seen from Table 6 that thermal comfort indexes of all working conditions are within the standard range (−1 ≤ PMV ≤1, PPD ≤ 27%), indicating that indoor thermal comfort is better under the three air conditioning operating modes proposed in this study. However, by comparing different modes, it is found that indoor comfort with both radiation and fresh air opened at the same time is better than that with radiation or fresh air opened alone. The PMV value and the PPD value are 0.13 and 5.2, respectively, when radiation and fresh air are opened at the same time in summer under normal refrigeration conditions. However, the PMV value and the PPD value are 0.22 and 5.41, respectively, when only fresh air is turned on, and the PMV value and the PPD value are 0.25 and 5.59, respectively, when only radiation is turned on. Under normal heating conditions in winter, when radiation and fresh air are opened at the same time, the PMV value and the PPD value are −0.23 and 5.39, respectively. However, the PMV value and the PPD value are −0.27 and 5.61, respectively, when only fresh air is turned on, and the PMV value the PPD value are −0.34 and 5.87, respectively, when only radiation is turned on. The above comparison results are sufficient to show that indoor thermal comfort is the best when radiation and fresh air are opened at the same time, and it is the worst when only radiation is opened. However, the three operating modes can meet the requirements of indoor thermal comfort. To put it simply, in the composite air conditioning system of ceiling radiation combined with an independent fresh air system, the radiation system bears part of the sensible heat load of indoors, while the independent fresh air system bears part of the sensible heat load and all the latent heat load of indoors. The combination of the two systems can provide a good thermal comfortable environment for the indoors.

## 7. Conclusion

This paper focuses on the technology of direct-expansion ceiling radiation and independent fresh air coupled with air conditioning systems to carry out scientific research. The temperature distribution uniformity of the indoor vertical plane and the horizontal plane is mainly studied when the radiation and fresh air operation modes are turned on at the same time. In addition, a detailed comparative analysis of the dehumidification effect of the system under refrigeration and dehumidification conditions is carried out. Finally, according to the measured data, the indoor thermal comfort index PMV-PPD value under different modes is calculated. The conclusions reached are as follows:

1. This research system adopts the form of coupling of direct expansion ceiling radiation and independent fresh air. The air conditioning system is composed of a heat pump air conditioning host, a new fan, and a radiation terminal. It directly uses refrigerant as a heat transfer medium to heat or refrigerate the air, and the new fan adopts direct expansion refrigeration dehumidification.
2. The system adopts a fin heat exchanger with the direct expansion of refrigerant. The large temperature difference between cooling and heating can be realized by the inline arrangement of finned metal radiation plates. Therefore, the system we designed not only has the characteristics of large heat flow per unit area, sufficient cooling capacity, and a simplified heat exchange system but also has the installation area of the finned metal radiation plate that only accounts for 14% of the ceiling.
3. The indoor temperature field test results show that the maximum temperature difference at the same time in the room is less than 2.1°C when radiation and fresh air mode are coupled under refrigeration conditions, and the maximum temperature difference at the same time in the room is less than 3°C when radiation and fresh air mode are coupled under heating condition. In general, the indoor temperature field is uniformly distributed when radiation and fresh air mode are coupled.
4. The dehumidification ability test of the system shows that the fresh air moisture content after dehumidification is between 5.48 g/kg and 9.63 g/kg, and the average dehumidification efficiency is more than 50%, which proves that the fresh air mode has obvious dehumidification effect.
5. The indoor thermal comfort index PMV and PPD calculated based on the experimental test data are −0.34 ≤ PMV ≤ 0.54 and 5.2 ≤ PPD ≤ 5.87, indicating that the indoor thermal comfort of the system designed in this study is relatively good.

The above research results show that the direct-expansion radiation and fresh air coupling air conditioning system proposed in this paper can realize independent control of temperature and humidity, which is very suitable for office and home use and has a good application prospect. In addition, the large temperature difference between refrigeration and heating radiation technology applied in the system, especially the direct-expansion radiative heat transfer technology, has certain novelty and creativity. However, there are still many technical and theoretical problems that need to be further improved to fully transform these technologies into practical engineering applications. In the future, further research can be done in the following areas to solve more problems that are scientific.

- Relevant researchers can carry out research on innovative air-conditioning technology and theoretical system based on large temperature differences and strong cold and heat radiation, especially on the mechanism of a large temperature difference and strong cold and heat radiation to achieve a uniform temperature of indoor radiation surface.
- With the support of the theory of heat transfer and computational fluid dynamics, scholars can carry out the simulation of heat and mass transfer in a complex thermal radiation environment.
- The subsequent related research work can focus on establishing the numerical analysis model of temperature field and airflow field of indoor thermal environment, which can provide a strong theoretical basis and application reference for the design standards, equipment manufacturing standards, and installation specifications related to strong cold and heat uniform radiation and direct expansion radiation air conditioning in the future.

## Figures and Tables

**Table 1:** Fin radiation plate parameters.

Copper pipe		Radiator fan	
Material	Cold drawn tubes	Material	Hydrophilic aluminum
Length	1,650 mm	Area	350 mm × 34 mm
Thermal conductivity	379.14 W/(m.K)	Thermal conductivity	237 W/(m.K)
Diameter	3/8” × *δ*0.7	Thickness	0.105 mm
Bay number	Single row	Spacing	1.8 mm
Quantity	16	Quantity	841

**Table 2:** Experimental instrument parameters used in this study.

Apparatus	Measuring range	Accuracy of measurement
Multichannel paperless recorder	Temperature: −50°C∼400°C; Humidity: 0∼100%RH	0.1/0.1%
Temperature and humidity transmitter	Temperature: −20°C∼80°C; Humidity: 0∼100%RH	±0.4/±3%
PT100 temperature sensor	−200°C∼200°C	0.5
Temperature and humidity recorder	Temperature: −20°C∼70°C; Humidity: 0∼100%RH	0.1
Anemometer	0∼30 m/s	±0.015

**Table 3:** Test point location parameters.

Plane *Z* = 0.1	Coordinates	Plane *Z* = 1.1	Coordinates	Plane *Z* = 1.7	Coordinates
A1	(1, 2.8, 0.1)	B1	(1, 2.8, 1.1)	C1	(1, 2.8, 1.7)
A2	(1, 1.1, 0.1)	B2	(1, 1.1, 1.1)	C2	(1, 1.1, 1.7)
A3	(3, 1.1, 0.1)	B3	(3, 1.1, 1.1)	C3	(3, 1.1, 1.7)
A4	(3, 2.8, 0.1)	B4	(3, 2.8, 1.1)	C4	(3, 2.8, 1.7)
A5	(2, 1.9, 0.1)	B5	(2, 1.9, 1.1)	C5	(2, 1.9, 1.7)

**Table 4:** Experimental design of radiation accompanying fresh air system.

	Outdoor temperature (°C)	Outdoor relative humidity (RH; %)	Indoor setting temperature (°C)
Low-temperature heating	−10∼−6	30	26
Ordinary heating	1∼5	30	

Ordinary refrigeration	28∼30	75	25
High-temperature cooling	37∼39	80	

**Table 5:** Moisture content of outdoor air and indoor fresh air.

Three kinds of working condition	Air samples	Average temperature (°C)	Average relative humidity (%)	Moisture content (g/kg)	Dehumidification efficiency (%)
Dehumidification in transition season (20°C∼22°C)	Air from the indoor air outlet	13.6	56.80	5.48	51.6
	Outdoor air	21.5	70.69	11.33	

Ordinary refrigeration (31°C∼33°C)	Air from the indoor air outlet	18.4	67.5	8.9	61.2
	Outdoor air	32.2	74.8	22.91	

High-temperature cooling (37°C∼39°C)	Air from the indoor air outlet	18.6	72.1	9.63	70.4
	Outdoor air	37.5	78.2	32.6	

**Table 6:** Indoor thermal comfort index test results.

Air conditioner operating mode	PMV-PPD value	General refrigeration (28°C∼30°C)	High-temperature refrigeration (37°C∼39°C)	Common heating (11°C∼15°C)	Low-temperature heating (−5°C∼−1°C)
Radiation	PMV	0.25	0.54	−0.34	−0.45
	PPD	5.59	7.17	5.87	6.21

Fresh air	PMV	0.22	0.49	−0.27	-0.4
	PPD	5.41	6.91	5.61	5.94

Radiation and fresh air at the same time	PMV	0.13	0.47	−0.23	−0.38
	PPD	5.2	6.82	5.39	5.75

## Data Availability

All data included in this study can be obtained from the corresponding author upon request.
